# The complete mitochondrial genome of freshwater mussel *Nodularia douglasiae* (Unionidae) from Lake Kasumigaura, Japan, and its phylogenetic analysis

**DOI:** 10.1080/23802359.2019.1674738

**Published:** 2019-10-11

**Authors:** Yohei Fukata, Masayuki Iigo

**Affiliations:** a Department of Applied Biological Chemistry, School of Agriculture, Utsunomiya University, Tochigi, Japan;; b Department of Biotechnology, United Graduate School of Agricultural Science, Tokyo University of Agriculture and Technology, Tokyo, Japan;; c Center for Bioscience Research and Education, Utsunomiya University, Tochigi, Japan;; d Center for Optical Research and Education, Utsunomiya University, Tochigi, Japan;; e Center for Weed and Wildlife Management, Utsunomiya University, Tochigi, Japan

**Keywords:** *Nodularia douglasiae*, Unioninae, freshwater mussel, Illumina sequence, molecular phylogenetic analysis

## Abstract

We have sequenced the female type (F-type) mitochondrial genome of *Nodularia douglasiae* (Unioninae, Unionidae, Unionida, Bivalvia) from Lake Kasumigaura, Japan, and inferred the Unioninae phylogeny using complete mitochondrial genome sequences. The complete F-type mitochondrial genome (15,779 bp; LC496352) contains 13 protein-coding genes, 2 rRNA genes, and 22 tRNA genes. Molecular phylogenetic analyses using complete F-type mitochondrial genomes from 15 Unioninae species including *N. douglasiae* from China and Korea were performed. This study should be basic data to investigate the genetic diversity of freshwater mussel *N. douglasiae.*

The freshwater mussels, the family Unionidae, are benthic bivalves that inhabit rivers and lakes widely. In the last decade, the number of Unionidae has been steadily decreasing. Globally, there is a concern about the deterioration of the habitat of the mussel bivalves (Lydeard et al. 2004). However, the cause of decrease in the habitat of bivalves and in population is not understood well (Lopes-Lima et al. 2017).

In Japan, 18 Unionidae species have been reported (Sano et al. 2017). Among them, *Nodularia. douglasiae* (Unioninae) inhabit not only in Japan but also in China, Korea, and eastern Russia (Liu et al. 2017). To reveal the phylogenetic position of the Japanese population of *N. douglasiae*, in this study, we sequenced the female-type (F-type) complete mitochondrial genome of *N. douglasiae* collected at Lake Kasumigaura, Japan, and molecular phylogenetic analyses were performed using complete F-type mitochondrial genomes of 15 Unioninae species including *N. douglasiae* from China and Korea.

*Nodularia douglasiae* (#UU-SBD-Unio-08) was collected at Lake Kasumigaura, Japan (N35.992, E140.349). Genomic DNA was extracted from the mantle using DNeasy Blood and Tissue Kit (QIAGEN, Valencia, CA, USA). Libraries for next-generation sequencing were prepared using TruSeq DNA PCR-Free Library Preparation Kit (Illumina, San Diego, CA, USA), Nextera XT DNA Library Preparation Kit (Illumina), and KAPA HyperPlus Kit (Kapa Biosystems, Wilmington, MA, USA). The nucleotide sequence was determined by 301 bp × 2 pair-end sequence using MiSeq (Illumina). Trimming and de novo sequence assembly were performed using CLC GenomicsWorkbench (ver 11.0.1). Local BLAST search using complete mitochondrial genome of *N. douglasiae* from Korea (NC_040162) identified the complete mitochondrial genome of *N. douglasiae* from Lake Kasumigaura, Japan (circular, 15779 bp). The results of BLAST search at NCBI (https://blast.ncbi.nlm.nih.gov/Blast.cgi) showed that the mitochondrial genome is the F-type. MITOS (http://mitos.bioinf.uni-leipzig.de/index.py; Bernt et al. 2013) and manual annotation predicted 13 protein-coding genes, 2 rRNA genes, and 22 tRNAs. The sequence has been submitted to DDBJ/EMBL/Genbank with an accession number of LC496352.

Molecular phylogenetic analysis was performed using the complete mitochondrial genomes of 15 Unioninae species. The sequences were aligned by ClustalW and a molecular phylogenetic tree was constructed by maximum-likelihood method (ML) using MEGA X (Kumar et al. 2018). Bootstrap analysis was performed with 1000 replications. As shown in Figure 1, the molecular phylogenetic tree formed two major clades. *N. douglasiae* is the closest to *Cuneopsis pisciculus* (NC_026306). The tree also showed that *N. douglasiae* from Japan (LC496352) is the sister to the clade of *N. douglasiae* from China (NC_026111) and Korea (MF314443), indicating the Japanese population diverged from the Eurasian group before the divergence of Chinese and Korean populations. This research would provide valuable information for investigating the genetic diversity of local populations of *N. douglasiae*.

**Figure 1.**
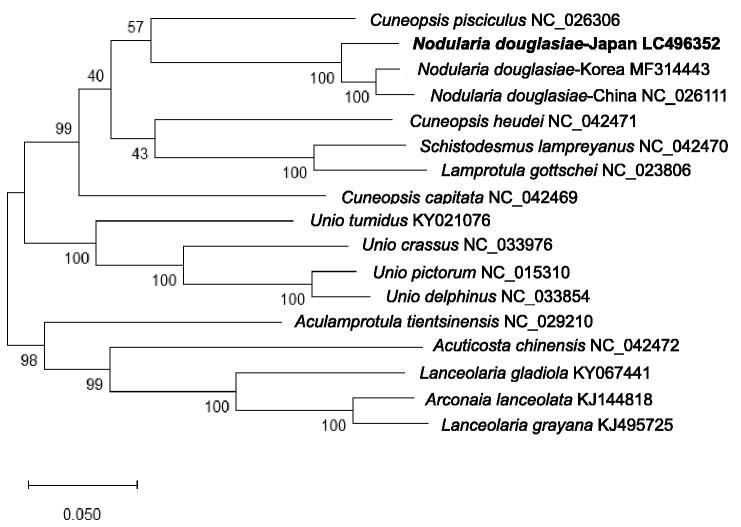
Molecular phylogenetic tree of 15 Unioninae species including *N. douglasiae* from Japan, China and Korea. Maximum-likelihood method was used to construct the tree using F-type complete mitochondrial genomes. The numbers above the branch meant bootstrap value (1000 replicates). Leaf names were presented as species names and accession number.
